# Supplementary material for: Microbiota Phenotype Promotes Anastomotic Leakage in a Model of Rats with Ischemic Colon Resection
Source: Microorganisms. 2023 Mar 7;11(3):680. doi: 10.3390/microorganisms11030680 (PMC10054737; doi:10.3390/microorganisms11030680)
Supplement: Supplementary file 1 [file microorganisms-11-00680-s001.zip › microorganisms-2165074-supplementary.pdf]

Table S1. Sequences, OTUs and OPUs. "Nr OPUs filtered" excluded identical values (i.e. zeros) across all samples and features that appear in only one sample. M: control mucosa samples, A: experimental anastomosis samples.

Sample	Nr total sequences	Nr OTUs	Nr OPUs	Nr OPUs filtered
**M1**	15387	132	44	12
**M2**	10213	47	15	12
**M3**	10659	149	75	67
**M4**	5943	65	37	37
**M5**	15444	171	83	72
**M6**	15606	142	56	49
**M7**	9237	132	75	64
**M8**	26448	564	13	12
**M9**	20689	379	1	1
**M10**	15960	224	40	34
**M11**	15328	346	18	11
**M12**	10774	66	9	8
**total**	171688	1569	210	124
**Mean**	**14307**	**201**	**39**	**32**
**SD**	5513	154	28	26
**A1**	12066	118	49	49
**A2**	12960	96	42	40
**A3**	11426	122	63	55
**A4**	12375	68	39	37
**A5**	16833	156	44	39
**A6**	13402	138	57	54
**A7**	16777	155	48	47
**A8**	18107	338	140	108
**A9**	17998	157	43	40
**A10**	14334	170	50	46
**A11**	12944	110	45	43
**A12**	18723	110	21	21
**total**	177945	1090	213	151
**Mean**	**14829**	**145**	**53**	**48**
**SD**	2668	68	29	21
**TOTAL**	**349633**	**2484**	**317**	**169**

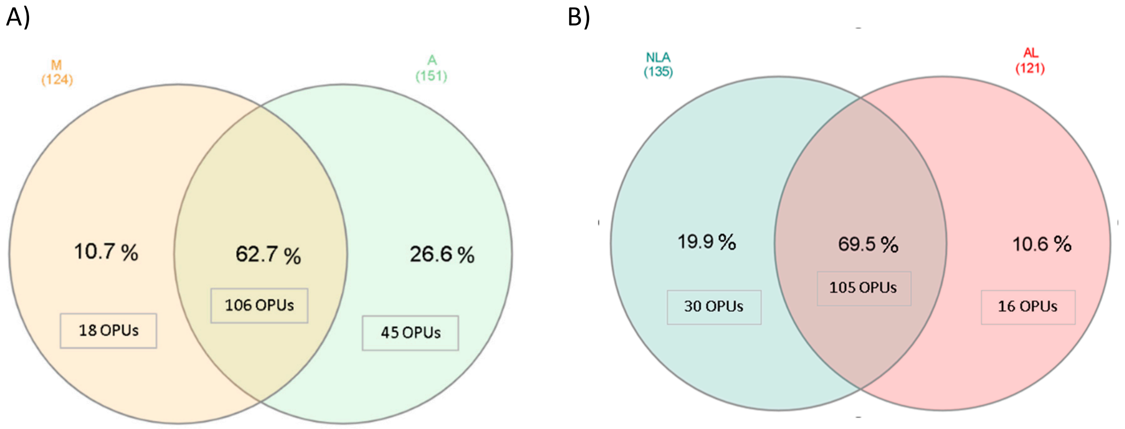

Figure S1. Venn diagram of OPUs obtained. These graphics were made after discarding OPUs with identical values in all samples, as well as OPUs present in only one sample. M: control mucosa group, A: experimental anastomosis group, NLA: non-leak anastomosis, AL: anastomotic leak.

Table S2. Alpha diversity indices. M: control mucosa samples, A: experimental anastomosis samples.

Sample	Chao-1	Dominance-D index	Shannon–Weiner index	Evenness
**M1**	44	0.07	3.01	0.46
**M2**	15	0.34	1.69	0.36
**M3**	75	0.09	3.07	0.29
**M4**	37	0.11	2.70	0.40
**M5**	83	0.06	3.38	0.36
**M6**	56	0.15	2.75	0.28
**M7**	75	0.09	3.06	0.29
**M8**	13	0.98	0.09	0.08
**M9**	1	1.00	0.00	1.00
**M10**	40	0.65	0.95	0.06
**M11**	18	0.81	0.58	0.10
**M12**	9	0.62	0.77	0.24
**Mean**	**39**	**0.41**	**1.84**	**0.33**
**SD**	28	0.37	1.29	0.25
**A1**	49	0.15	2.55	0.26
**A2**	42	0.10	2.73	0.37
**A3**	63	0.21	2.38	0.17
**A4**	39	0.18	2.41	0.29
**A5**	44	0.65	1.05	0.06
**A6**	57	0.10	2.81	0.29
**A7**	48	0.41	1.66	0.11
**A8**	140	0.04	3.79	0.32
**A9**	43	0.27	1.56	0.11
**A10**	50	0.47	1.35	0.08
**A11**	45	0.19	2.18	0.20
**A12**	21	0.21	1.90	0.32
**Mean**	**53**	**0.25**	**2.20**	**0.21**
**SD**	29	0.18	0.75	0.11

Table S3. Heat tree data of taxonomic differences between A and M. Result from comparison between microbial communities of Figure 3. Statistically, Wilcoxon Rank Sum test $p < 0.05$. M: control, A: anastomosis. "p"=phylum, "c"=class, "o"=order, "f"=family, "g"=genus, "s"=species.

tax_rank	tax_name	G1	G2	log2_median_ratio	median_diff	mean_diff	wilcox_p_value
p	*Fusobacteriota*	A	M	Inf	0.0056537	0.016737	0.000964
p	*Campylobacterota*	A	M	-6.775942901	-0.4878357	-0.491993	0.003407
p	*Firmicutes*	A	M	1.042100162	0.2089626	0.226212	0.020489
p	*Proteobacteria*	A	M	1.471870751	0.1571441	0.132335	0.026197
c	*Fusobacteriia*	A	M	Inf	0.0056537	0.016737	0.000964
c	*Campylobacteria*	A	M	-6.775942901	-0.4878357	-0.491993	0.003407
c	*Bacilli*	A	M	3.15323081	0.2624777	0.191711	0.017271
c	*Gammaproteobacteria*	A	M	1.462988704	0.1556358	0.136881	0.022459
c	*Negativicutes*	A	M	3.116655648	0.0477196	0.039915	0.039536
o	*Fusobacteriales*	A	M	Inf	0.0056537	0.016737	0.000964
o	*Rhodospirillales*	A	M	Inf	0.0005718	0.004350	0.001084
o	*Staphylococcales*	A	M	Inf	0.1879922	0.225497	0.001849
o	*Campylobacterales*	A	M	-6.775942901	-0.4878357	-0.491993	0.003407
o	*Acidaminococcales*	A	M	Inf	0.0146038	0.020108	0.003929
o	*Enterobacterales*	A	M	5.243994683	0.2377824	0.154125	0.005880
o	*RF39*	A	M	Inf	0.0006630	0.001313	0.030982
o	*Xanthomonadales*	A	M	0	0.0000000	-0.009254	0.036687
f	*Gemellaceae*	A	M	Inf	0.1879922	0.267173	0.000038
f	*Pasteurellaceae*	A	M	Inf	0.0806828	0.126894	0.000052
f	*Fusobacteriaceae*	A	M	Inf	0.0056537	0.016737	0.000964
f	*Helicobacteraceae*	A	M	-6.775942901	-0.4878357	-0.491993	0.003407
f	*Acidaminococcaceae*	A	M	Inf	0.0146038	0.020108	0.003929
f	*Bacteroidales_1*	A	M	Inf	0.0002320	0.001015	0.007054
f	*Bacteroidaceae*	A	M	5.618467188	0.1668760	0.154478	0.011654
f	*Rikenellaceae*	A	M	Inf	0.0013822	0.002167	0.013072
f	*Carnobacteriaceae*	A	M	Inf	0.0010055	0.005052	0.033976
f	*Streptococcaceae*	A	M	0	0.0000000	0.002554	0.036687
f	*Staphylococcaceae*	A	M	0	0.0000000	-0.041676	0.036687
f	*Xanthomonadaceae*	A	M	0	0.0000000	-0.009254	0.036687
f	*Rhodospirillales_3*	A	M	0	0.0000000	0.001035	0.036687
f	*Rhodospirillales_4*	A	M	0	0.0000000	0.002979	0.036687
f	*RF39_5*	A	M	0	0.0000000	0.000542	0.036687
f	*RF39_6*	A	M	0	0.0000000	0.000529	0.036687
g	*Gemella*	A	M	Inf	0.1879922	0.267173	0.000038
g	*Rodentibacter*	A	M	Inf	0.0806828	0.126894	0.000052
g	*Fusobacterium*	A	M	Inf	0.0056537	0.016737	0.000964
g	*Helicobacter*	A	M	-6.775942901	-0.4878357	-0.491993	0.003407
g	*Phascolarctobacterium*	A	M	Inf	0.0146038	0.020108	0.003929
g	*Bacteroidales_1*	A	M	Inf	0.0002320	0.001015	0.007054
g	*Bacteroides*	A	M	5.618467188	0.1668760	0.154478	0.011654
g	*Prevotellaceae_1*	A	M	0	0.0000000	0.001209	0.016473
g	*Sphaerochaeta*	A	M	0	0.0000000	0.000586	0.016473
g	*UCG_008*	A	M	0	0.0000000	0.000422	0.016473
g	*Carnobacteriaceae_1*	A	M	Inf	0.0010055	0.005052	0.033976
g	*Facklamia*	A	M	0	0.0000000	-0.027586	0.036687
g	*Globicatella*	A	M	0	0.0000000	0.002705	0.036687
g	*Streptococcus*	A	M	0	0.0000000	0.002554	0.036687
g	*Jeotgalicoccus*	A	M	0	0.0000000	-0.011633	0.036687
g	*Xanthomonadaceae_1*	A	M	0	0.0000000	-0.009254	0.036687
g	*Rhodospirillales_3*	A	M	0	0.0000000	0.001035	0.036687
g	*Rhodospirillales_4*	A	M	0	0.0000000	0.002979	0.036687
g	*RF39_5*	A	M	0	0.0000000	0.000542	0.036687
g	*RF39_6*	A	M	0	0.0000000	0.000529	0.036687
g	*Johnsonella*	A	M	0	0.0000000	0.000219	0.036687
s	*Gemella_palaticanis*	A	M	Inf	0.1879922	0.267173	0.000038
s	*Rodentibacter_ratti*	A	M	Inf	0.0276695	0.088834	0.000079
s	*Bacteroides_sartorii*	A	M	Inf	0.0489763	0.074472	0.000505
s	*Fusobacterium_gastrosuis*	A	M	Inf	0.0056537	0.016737	0.000964
s	*Bacteroides_uniformis*	A	M	Inf	0.0009443	0.006443	0.001084
s	*Rodentibacter_4*	A	M	Inf	0.0009632	0.012824	0.002856
s	*Phascolarctbacterium_succinatutens*	A	M	Inf	0.0146038	0.020108	0.003929
s	*Bacteroidales_1*	A	M	Inf	0.0002320	0.001015	0.007054
s	*Rodentibacter_3*	A	M	Inf	0.0002707	0.011388	0.007054
s	*Prevotellaceae_1*	A	M	0	0.0000000	0.001209	0.016473
s	*Sphaerochaeta_1*	A	M	0	0.0000000	0.000586	0.016473
s	*Rodentibacter_6*	A	M	0	0.0000000	0.005194	0.016473
s	*UCG_008_1*	A	M	0	0.0000000	0.000422	0.016473
s	*Helicobacter_muridarum*	A	M	0	-0.0116239	-0.098321	0.028503
s	*Carnobacteriaceae_1*	A	M	Inf	0.0010055	0.005052	0.033976
s	*Facklamia_tabacinasalis*	A	M	0	0.0000000	-0.027586	0.036687
s	*Globicatella_1*	A	M	0	0.0000000	0.002705	0.036687
s	*Streptococcus_1*	A	M	0	0.0000000	0.002554	0.036687
s	*Jeotgalicoccus_nanhaiensis*	A	M	0	0.0000000	-0.011633	0.036687
s	*Alloprevotella_2*	A	M	0	0.0000000	0.000370	0.036687
s	*Escherichia_coli_4*	A	M	0	0.0000000	0.001599	0.036687
s	*Escherichia_Shigella_2*	A	M	0	0.0000000	0.003043	0.036687
s	*Rodentibacter_1*	A	M	0	0.0000000	0.009411	0.036687
s	*Xanthomonadaceae_1*	A	M	0	0.0000000	-0.009254	0.036687
s	*Rhodospirillales_3*	A	M	0	0.0000000	0.001035	0.036687
s	*Rhodospirillales_4*	A	M	0	0.0000000	0.002979	0.036687
s	*RF39_5*	A	M	0	0.0000000	0.000542	0.036687
s	*RF39_6*	A	M	0	0.0000000	0.000529	0.036687
s	*Johnsonella_1*	A	M	0	0.0000000	0.000219	0.036687
s	*Oribacterium_1*	A	M	0	0.0000000	0.000514	0.036687
s	*Rikenellaceae_RC9_gut_group_3*	A	M	Inf	0.0005239	0.001617	0.040513
s	*Helicobacter_1*	A	M	0	-0.0208354	-0.311105	0.041607
s	*Bacteroides_acidifaciens*	A	M	Inf	0.0049116	0.007562	0.048481

Table S4. Data summary of LEfSe analysis between A and M. Data of taxonomic differences between A and M from Figure 5A. LEfSe/LDA score >2, and a raw P value cutoff of 0.05, Kruskal-Wallis test. M: control, A: anastomosis.

OPU	Species	P values	FDR	A	M	LDA score
OPU_138	*Gemella_palaticanis*	0.00003	**0.005**	2672200	0	6.1
OPU_334	*Rodentibacter_ratti*	0.00007	**0.006**	890820	2366	5.7
OPU_172	*Bacteroides_sartorii*	0.00045	**0.025**	829660	84909	5.6
OPU_48	*Phascolarctbacterium_succinatutens*	0.00357	0.085	207440	6341.7	5.0
OPU_325	*Fusobacterium_gastrosuis*	0.00086	**0.032**	174210	6810.9	4.9
OPU_338	*Rodentibacter_4*	0.00254	0.070	128390	0	4.8
OPU_337	*Rodentibacter_3*	0.00629	0.116	114050	0	4.8
OPU_335	*Rodentibacter_1*	0.03287	0.182	94320	0	4.7
OPU_167	*Bacteroides_acidifaciens*	0.04513	0.227	100350	24660	4.6
OPU_170	*Bacteroides_uniformis*	0.00096	**0.032**	64426	0	4.5
OPU_340	*Rodentibacter_6*	0.01473	0.182	52015	0	4.4
OPU_122	*Carnobacteriaceae_1*	0.03106	0.182	53878	3356.4	4.4
OPU_332	*Escherichia_Shigella_2*	0.03287	0.182	30448	0	4.2
OPU_358	*Rhodospirillales_4*	0.03287	0.182	29812	0	4.2
OPU_126	*Globicatella_1*	0.03287	0.182	27047	0	4.1
OPU_127	*Streptococcus_1*	0.03287	0.182	25539	0	4.1
OPU_314	*Rikenellaceae_RC9_gut_group_3*	0.03712	0.199	17960	1783.4	3.9
OPU_330	*Escherichia_coli_4*	0.03287	0.182	16006	0	3.9
OPU_157	*Prevotellaceae_1*	0.01473	0.182	12113	0	3.8
OPU_308	*Bacteroidales_1*	0.00629	0.116	10152	0	3.7
OPU_357	*Rhodospirillales_3*	0.03287	0.182	10349	0	3.7
OPU_323	*Sphaerochaeta_1*	0.01473	0.182	5860.9	0	3.5
OPU_383	*RF39_5*	0.03287	0.182	5424.5	0	3.4
OPU_384	*RF39_6*	0.03287	0.182	5294.2	0	3.4
OPU_94	*Oribacterium_1*	0.03287	0.182	5146.6	0	3.4
OPU_37	*UCG_008_1*	0.01473	0.182	4218.3	0	3.3
OPU_179	*Alloprevotella_2*	0.03287	0.182	3702	0	3.3
OPU_91	*Johnsonella_1*	0.03287	0.182	2186.2	0	3.0
OPU_353	*Xanthomonadaceae_1*	0.03287	0.182	0	97310	-4.7
OPU_137	*Jeotgalicoccus_nanhaiensis*	0.03287	0.182	0	123270	-4.8
OPU_125	*Facklamia_tabacinasalis*	0.03287	0.182	0	292480	-5.2
OPU_362	*Helicobacter_muridarum*	0.02634	0.182	25595	1011500	-5.7
OPU_361	*Helicobacter_1*	0.03870	0.201	71139	3195300	-6.2

Table S5. Correlation coefficients results between A and M. Correlation values in Figure 5B are shown. Taxon 1 correspond to species highlighted in Figure 5, with a LEfSe-LDA score >2 and a FDR-adjusted P value < 0.05. Taxon 2 correspond to species with LEfSe/LDA score >2, and a raw P value cutoff < 0.05, Kruskal-Wallis test.

**Taxon 1**	**Taxon 2**	**Correlation**	**P Value**
Gemella_palaticanis	*Rodentibacter_ratti*	0.8373	0.0099
Gemella_palaticanis	*Bacteroides_uniformis*	0.561	0.0198
Gemella_palaticanis	*Rodentibacter_4*	0.4474	0.0495
Gemella_palaticanis	*Phascolarctbacterium_succinatutens*	0.4079	0.0198
Gemella_palaticanis	*Jeotgalicoccus_nanhaiensis*	-0.4422	0.0198
Gemella_palaticanis	*Facklamia_tabacinasalis*	-0.4685	0.0297
Gemella_palaticanis	*Xanthomonadaceae_1*	-0.469	0.0297
Gemella_palaticanis	*Helicobacter_muridarum*	-0.5158	0.0099
Rodentibacter_ratti	*Gemella_palaticanis*	0.8373	0.0099
Rodentibacter_ratti	*Bacteroides_uniformis*	0.5348	0.0198
Rodentibacter_ratti	*Fusobacterium_gastrosuis*	0.412	0.0396
Rodentibacter_ratti	*Rodentibacter_4*	0.4013	0.0198
Rodentibacter_ratti	*Helicobacter_muridarum*	-0.445	0.0297
Bacteroides_sartorii	*Bacteroides_acidifaciens*	0.6415	0.0099
Bacteroides_sartorii	*Helicobacter_1*	-0.3869	0.0495
Fusobacterium_gastrosuis	*Rodentibacter_ratti*	0.412	0.0396
Fusobacterium_gastrosuis	*Helicobacter_1*	-0.3983	0.0297
Bacteroides_uniformis	*Gemella_palaticanis*	0.561	0.0198
Bacteroides_uniformis	*Rodentibacter_ratti*	0.5348	0.0198
Bacteroides_uniformis	*Rodentibacter_4*	0.4743	0.0297
Bacteroides_uniformis	*RF39_5*	-0.412	0.0495
Bacteroides_uniformis	*Rodentibacter_1*	-0.4128	0.0297
Bacteroides_uniformis	*UCG_008_1*	-0.4378	0.0297
Bacteroides_uniformis	*Helicobacter_muridarum*	-0.5107	0.0099

Table S6. Microbial respiration of microbiota. A: anastomosis experimental group, M: control group.

Microbial respiration of microbiota, relative abundances (%)																								
	A01	A02	A03	A04	A05	A06	A07	A08	A09	A10	A11	A12	M01	M02	M03	M04	M05	M06	M07	M08	M09	M10	M11	M12
Aerobes	0.0	0.0	0.0	0.0	0.0	0.0	0.0	0.0	0.0	0.0	0.0	0.0	23.2	11.5	0.0	0.0	1.5	0.4	0.0	0.1	0.0	0.0	0.0	0.0
Facultative anaerobes	9.4	58.3	57.6	37.0	82.5	36.8	66.3	47.8	56.3	20.4	47.3	71.4	65.4	22.5	15.1	19.2	19.6	10.7	12.1	0.4	0.0	1.8	1.3	0.0
Obligate anaerobes	90.0	41.7	35.6	56.2	13.4	59.0	32.8	39.6	43.1	78.9	44.5	28.6	0.8	0.5	82.8	80.8	64.6	53.2	54.7	0.3	0.0	4.8	2.7	0.8
Microaerophiles	0.2	0.0	2.5	6.4	3.3	3.4	0.7	11.0	0.1	0.3	7.5	0.0	10.7	65.4	1.7	0.0	13.4	35.5	27.0	99.2	100.0	92.9	95.5	99.2
microaerophile or aerotolerant anaerobes	0.0	0.0	4.0	0.4	0.7	0.6	0.2	0.9	0.4	0.0	0.1	0.0	0.0	0.0	0.0	0.0	0.0	0.3	4.6	0.0	0.0	0.3	0.5	0.0
Microaerophiles or facultatively anaerobic	0.4	0.0	0.0	0.0	0.0	0.0	0.0	0.2	0.0	0.0	0.0	0.0	0.0	0.0	0.2	0.0	0.9	0.0	1.5	0.0	0.0	0.2	0.0	0.0
ND	0.0	0.0	0.3	0.0	0.1	0.2	0.0	0.4	0.1	0.4	0.6	0.0	0.0	0.0	0.1	0.0	0.0	0.0	0.1	0.0	0.0	0.0	0.0	0.0

Microbial respiration of microbiota, relative abundances (Log10)																								
	A01	A02	A03	A04	A05	A06	A07	A08	A09	A10	A11	A12	M01	M02	M03	M04	M05	M06	M07	M08	M09	M10	M11	M12
Aerobes	0.0	0.0	0.0	0.0	0.0	0.0	0.0	0.0	0.0	0.0	0.0	0.0	1.4	1.1	0.0	0.0	0.4	0.1	0.0	0.0	0.0	0.0	0.0	0.0
Facultative anaerobes	1.0	1.8	1.8	1.6	1.9	1.6	1.8	1.7	1.8	1.3	1.7	1.9	1.8	1.4	1.2	1.3	1.3	1.1	1.1	0.1	0.0	0.4	0.4	0.0
Obligate anaerobes	2.0	1.6	1.6	1.8	1.2	1.8	1.5	1.6	1.6	1.9	1.7	1.5	0.2	0.2	1.9	1.9	1.8	1.7	1.7	0.1	0.0	0.8	0.6	0.2
Microaerophiles	0.1	0.0	0.5	0.9	0.6	0.6	0.2	1.1	0.0	0.1	0.9	0.0	1.1	1.8	0.4	0.0	1.2	1.6	1.4	2.0	2.0	2.0	2.0	2.0
Microaerophile or aerotolerant anaerobes	0.0	0.0	0.7	0.1	0.2	0.2	0.1	0.3	0.1	0.0	0.0	0.0	0.0	0.0	0.0	0.0	0.0	0.1	0.7	0.0	0.0	0.1	0.2	0.0
Microaerophiles or facultatively anaerobic	0.2	0.0	0.0	0.0	0.0	0.0	0.0	0.1	0.0	0.0	0.0	0.0	0.0	0.0	0.1	0.0	0.3	0.0	0.4	0.0	0.0	0.1	0.0	0.0
ND	0.0	0.0	0.1	0.0	0.1	0.1	0.0	0.1	0.0	0.1	0.2	0.0	0.0	0.0	0.1	0.0	0.0	0.0	0.1	0.0	0.0	0.0	0.0	0.0

Table S7. Data summary of LEfSe analysis between NLA and AL. Data of taxonomic differences between NLA and AL from Figure 8B. LEfSe/LDA score >2, and a raw P value cutoff of 0.05, Kruskal-Wallis test. NLA: non-leak anastomosis, AL: anastomotic leak.

**OPU**	**Species**	**P values**	**FDR**	**AL**	**NLA**	**LDA score**
OPU_365	*Helicobacter_rodentium*	0.037222	0.45694	3170.9	78507	4.58
OPU_114	*Lactobacillus_murinus*	0.020122	0.45694	882.53	45697	4.35
OPU_116	*Ligilactobacillus_1*	0.036714	0.45694	0	31283	4.19
OPU_104	*Lactobacillus_reuteri*	0.036714	0.45694	0	16714	3.92
OPU_355	*Rhodospirillales_1*	0.036714	0.45694	0	10369	3.71
OPU_31	*UCG_005_6*	0.036714	0.45694	0	9376.8	3.67
OPU_145	*Clostridium_sensu_stricto_1_2*	0.036714	0.45694	0	7009.5	3.54
OPU_199	*Muribaculaceae_20*	0.036714	0.45694	0	1826.1	2.96
OPU_209	*Muribaculaceae_30*	0.036714	0.45694	0	1159.4	2.76
OPU_113	*Lactobacillus_2*	0.038015	0.45694	51288	45881	-3.43
OPU_138	*Gemella_palaticanis*	0.04154	0.45694	3568600	893270	-6.13

Table S8. Correlation coefficient results between NLA and AL. Correlation values in Figure 8C are shown. LEfSe-LDA score >2 and a raw P value < 0.05, Kruskal-Wallis test.

**Taxon 1**	**Taxon 2**	**Correlation**	**P Value**
Gemella_palaticanis	*Helicobacter_rodentium*	-0.6012	0.0099
Clostridium_sensu_stricto_1_2	*Muribaculaceae_30*	0.9367	0.0099
Ligilactobacillus_1	*Lactobacillus_marinus*	0.6419	0.0396
Lactobacillus_marinus	*Rhodospirillales_1*	0.5738	0.0396
Rhodospirillales_1	*Muribaculaceae_20*	0.9122	0.0099
Rhodospirillales_1	UCG_005_6	0.926	0.0099
Muribaculaceae_20	UCG_005_6	0.9034	0.0099
